# Pediatric Intestinal Pseudo-Obstruction: Progress and Challenges

**DOI:** 10.3389/fped.2022.837462

**Published:** 2022-04-13

**Authors:** Marie-Catherine Turcotte, Christophe Faure

**Affiliations:** Division of Pediatric Gastroenterology, Hepatology and Nutrition, Sainte-Justine University Health Centre, Université de Montréal, Montreal, QC, Canada

**Keywords:** Pediatric Intestinal Pseudo-Obstruction, myopathy, neuropathy, autoimmune GI dysmotility, antroduodenal manometry, intestinal transplantation

## Abstract

**Background:**

Chronic intestinal pseudo-obstruction is a rare disorder and represents the most severe form of gastrointestinal dysmotility with significant morbidity and mortality. Emerging research shows considerable differences between the adult and pediatric population with intestinal pseudo-obstruction and the term Pediatric Intestinal Pseudo-Obstruction (PIPO) was recently proposed.

**Purpose:**

The aim of this article is to provide pediatric gastroenterologists and pediatricians with an up to date review of the etiology and underlining pathophysiology, clinical features, diagnostic and management approaches currently available for PIPO and to discuss future perspectives for the diagnosis and management of this rare disease.

## Introduction

Chronic intestinal pseudo-obstruction (CIPO) is “a rare, severe, disabling disorder characterized by repetitive episodes or continuous symptoms and signs of bowel obstruction, including radiographic documentation of dilated bowel with air fluid levels, in the absence of a fixed, lumen-occluding lesion” (1). It represents a group of heterogeneous disorders and can affect any part of the gastrointestinal (GI) tract with the small intestine and colon mostly affected. CIPO is regarded as one of the most severe form of gut dysmotility and the prognosis in children remains guarded even with the advent of parental nutrition and intestinal transplantation (2, 3). The mortality is estimated between 10 and 32% with a significant morbidity for the remaining (4), but the prognosis of these patients is not clear. Studies from France and the United States found that the course is more severe in the pediatric population with 60–80% needing parental nutrition and 10–25% dying before adulthood (5, 6). Interestingly, a recent survey in Japan revealed that only 10% of the patients needed total parental nutrition and only 4% died from enteritis and sepsis with no statistically significant difference between the neonatal-onset and the post-neonatal onset groups (7).

Even though the term CIPO was applied to adult and pediatric population, emerging research shows considerable differences between these two groups with more congenital and primary forms of CIPO in the pediatric population. Consequently, the latest consensus proposed the term Pediatric Intestinal Pseudo-Obstruction (PIPO) for pediatric patients with CIPO (2). Being a rare disease, PIPO poses a clinical challenge and the diagnostic is often not recognized or mistaken for another functional GI disorder. Therefore, acutely estimating the prevalence of PIPO is difficult. A recent nationwide survey in Japan reported a prevalence of 3.7 in one million children with equal sex incidence (7). A nationwide study in the United States stated that around 100 infants were born every year with PIPO (8) and a more recent United States nationwide survey found an incidence of inpatient admission of 29 per 100,000 patients (9). Even though rare, PIPO inpatient admissions carry a high healthcare burden and the presence of malnutrition, parental nutrition and its complications result in longer duration and higher cost of hospitalization (9).

Pediatric Intestinal Pseudo-Obstruction remains an under-diagnosed condition and there are a lot of potential areas for future research. The objective of this review is to provide a succinct outline of the etiology and underlining pathophysiology, clinical features, diagnostic and treatments currently available for PIPO in 2022.

## Etiology and Pathophysiology

The etiology of PIPO can be either primary (sporadic or familial), secondary or idiopathic. As stated before, children are affected predominantly by primary disorders of enteric neuromusculature, either by inflammation degradation or abnormal development of the enteric neuromusculature (10) and can incorporate neuropathy, myopathy, or mesenchymopathy (11). They can be further classified depending on where the insult is: enteric neurons, intestinal smooth muscle or the interstitial cells of Cajal (ICC) network (10). Primary PIPO also include inflammatory (including autoimmune) conditions as lymphocytic and eosinophilic ganglionitis and/or leiomyositis (2, 10). It also includes mitochondrial neuro-gastrointestinal-encephalomyopathy (MNGIE) and other mitochondrial diseases and neuropathy associated with multiple endocrine neoplasia type IIB (2).

Autoimmune enteric leiomyositis typically presents in infancy or early childhood with elevated antibodies on laboratory findings. Histopathology usually shows lymphocytic infiltrate of the muscularis propria on full-thickness biopsies of the small intestine (10, 12).

Patients with eosinophilic myenteric ganglionitis typically presents in the neonatal or childhood period and have histopathological findings of eosinophilic infiltration within the myenteric plexus, submucosa, and mucosa on full-thickness biopsies of the small intestine (10, 13).

With the advancement of genetic sequencing, multiple genetic mutations have been identified and associated with PIPO (Table 1; 10).

**TABLE 1:** Monogenic mutations associated with Pediatric Intestinal Pseudo-Obstruction.

Gene	Syndrome	Age of onset
Sox 10	Type IV Waardenburg syndrome	Neonatal period
POLG1	Congenital myopathy and GI pseudo-obstruction	Neonatal period
FLNA	Chronic idiopathic intestinal pseudo-obstruction	Neonatal period
L1CAM	Hydrocephalus with stenosis of aqueduct of Sylvius and congenital idiopathic intestinal pseudo-obstruction	Neonatal period
ACTG2	Familial visceral myopathy; megacystis-microcolon-intestinal hypoperistaltism syndrome (MMIHS)	Neonatal–3rd decade in life
MYH11	MMIHS	Neonatal–3rd decade in life
MYLK	MMIHS	Neonatal–3rd decade in life
LMOD1	MMIHS	Neonatal–3rd decade in life
MYL9	MMIHS	Neonatal–3rd decade in life
RET proto-oncogene	MEN2B	Infancy–3rd decade in life
TYMP	MNGIE	Infancy–3rd decade in life
RAD21	Mungan syndrome	1st–2nd decade in life
SGOL1	Chronic atrial and intestinal dysrhythmia	1st–4th decade in life

Secondary causes of PIPO are listed in Table 2 and includes conditions affecting GI smooth muscle, pathologies affecting the enteric nervous system (ENS), endocrinological disorders, metabolic conditions, toxic causes, immune diseases, and others (2, 14). Hirschsprung disease needs to be excluded in all forms of PIPO.

**TABLE 2:** Secondary causes of Pediatric Intestinal Pseudo-Obstruction.

CONDITIONS AFFECTING GI SMOOTH MUSCLE	ENDOCRINOLOGICAL DISORDERS
**Rheumatologic conditions:** Dermatomyositis/polymyositis, scleroderma, systemic lupus erythematosus, Ehler Danlos syndrome	Hypothyroidism, diabetes, hypoparathyroidism, pheochromocytoma
**Other:** Duchenne muscular dystrophy, myotonic dystrophy, amyloidosis, ceroidosis	**METABOLIC CONDITIONS** Uremia, porphyria, electrolyte imbalances (potassium, magnesium, calcium), carnitine deficiency, vitamin E deficiency
**PATHOLOGIES AFFECTING THE ENTERIC NERVOUS SYSTEM**	**TOXIC**
Familial dysautonomia, primary dysfunction of the autonomic nervous system, autoimmune GI dysmotility, neurofibromatosis, diabetic neuropathy, fetal alcohol syndrome **Post-viral related inflammatory neuropathy** (54): CMV, EBV, VZV, John Cunningham virus (JCV) virus, HSV, Flaviviruses, Lyme disease	Ketamine, Carbamazepine, Clonidine, Atropine, Fludarabin, Vinplastin, Vincristin, neuroleptics, antidepressants, Phenothiazine, opiates, calcium channel blockers
	**OTHER**
	Coeliac disease, Crohn disease, eosinophilic gastroenteritis, radiation injury, paraneoplasic syndrome, Chagas disease, Kawasaki disease, angio-oedema

Autoimmune GI dysmotility (AGID) is a manifestation of autoimmune dysautonomia, affecting the ENS, that can either be idiopathic or secondary to a paraneoplasic syndrome (15). Different neuronal autoantibodies have been identified: anti-ganglionic type acetylcholine nicotinic receptors (AChR), anti-voltage-gated potassium channels (anti-VGKC), anti-voltage-gated calcium channels (anti-VGCC), type 1 anti-neuronal nuclear antibody (ANNA-1 or anti-Hu), anti-muscle AChR, anti-GAD65, anti-peripherin, and anti-Yo antibodies (15, 16). Anti-Hu GI dysmotility commonly occurs as a paraneoplasic syndrome (neuroblastoma in children), but several cases of non-paranoeplasic cases have been reported, especially in younger patients with a longer course of disease (16). Other neurological autoimmune disorders can present with early GI symptoms and are important to identify since they can greatly improve with immunotherapy. These include antibodies targeting the aquaporin-4 (AQP4) water channel, dipeptidyl-peptidas-like protein 6 (DPPX) antibodies and anti-CRP5 antibodies (17).

Idiopathic is defined when a primary or secondary etiology has not yet been identified.

## Clinical Features

The clinical presentation of PIPO depends on age at onset, the site and severity of the affected GI tract. Children with symptoms of intestinal obstruction without an occluding lesion should be suspected to have PIPO (2). In around 20% of cases, prenatal signs can be identified (6); megacystits being the most frequently reported sign. Surprisingly, dilated bowels are rarely identified during prenatal ultrasounds. Neonatal urological symptoms may come before GI symptoms after birth (6).

For 50–70% of the patients, clinical manifestations start in the first month of life, for 80% of the patients, in the first year. The others usually have a sporadic onset during their first 20 years of life (6, 18).

In the neonatal-onset form, the typical presentation consists of a combination of bilious or non-bilious vomiting, abdominal distention, and intestinal occlusion (19). In preterm infants, the diagnosis should be made with caution because the migrating motor complex (MMC) appears in its mature form only at 34–35 weeks of gestation. PIPO may then be mimicked by immature intestinal motility (20). Furthermore, the neonatal-onset form is significantly associated with urinary involvement as confirmed by a large retrospective study over a 30-year period (19).

In the late-onset form, symptoms depend on the part of the GI tract damaged. This form shares more clinical aspects with the adult CIPO than the neonatal form (4). The main symptoms are abdominal distention (88%), vomiting (69%), constipation (54%), failure to thrive (31%), abdominal pain, diarrhea (24%), and dysphagia (3%) (2). Various triggers may precipitate exacerbations including intercurrent infections, fever, general anesthesia, psychological stress, and malnutrition (2, 18). Some patients may also present with diarrhea and steatorrhea if small intestinal bacterial overgrowth (SIBO) occurs secondary to intestinal stasis (4). Late-onset PIPO is also associated with more comorbidities, except for malrotation, than the neonatal-onset PIPO (19).

Patients with suspected AGID can present with acute or subacute (<8 weeks) onset of GI symptoms, family history of autoimmune diseases, an infectious episode preceding the onset and extra-intestinal neurological symptoms like dysautonomia. They also tend to not improve with symptomatic treatment (15, 17).

Malabsorption and malnutrition are frequent since oral feedings are a challenge for PIPO patients. If parental nutrition has not started because the diagnosis is delayed, nutritional complications can arise from micronutrient deficiencies and malabsorption (10).

## Comorbidities

Intestinal malrotation is ten times more likely in children with congenital PIPO compared to normal children (6). The incidence seems to be the same in myopathic and neuropathic PIPO (5), but is more associated with the neonatal-onset form (19). In a large retrospective study over a 30-year period, Diamanti et al. recommended that PIPO should be suspected in the case of persistent vomiting after a Ladd’s procedure for malrotation (19). Malrotation has also been found in X-linked familial syndromes with PIPO, pyloric non-hypertrophic stenosis, and malrotation (21).

Urinary tract involvement happens in 33–92% of cases, depending on the type of PIPO (2). It occurs more commonly in those with congenital myopathy (6). The most frequent pattern of urological abnormality is megacystis with a hypocontractile detrusor, increased bladder capacity and compliance (22). Thus, a careful evaluation of bladder function is recommended.

Some patients may also present with dysmotility of the gallbladder and cholelithiasis (2, 19).

Finally, cardiomyopathy or dysfunction of cardiac sinus node may occur with PIPO so patients should be evaluated with ECG and echocardiography (2, 23).

## Diagnosis

The ESPGHAN published in 2018 the first expert consensus on PIPO and the following definition and diagnostic criteria: “PIPO is a disorder characterized by the chronic (≥2 months from birth or ≥6 months thereafter) inability of the GI tract to propel its contents mimicking mechanical obstruction, in the absence of any lesion occluding the gut” (2).

The diagnosis requires “at least 2 out of 4 of the following:

• Objective measure of small intestinal neuromuscular involvement (manometry, histopathology, transit).
• Recurrent and/or persistently dilated bowel loops of small intestine with air fluid levels.
• Genetic and/or metabolic abnormalities associated with PIPO.
• Inability to maintain adequate nutrition and/or growth on oral feeding (needing specialized enteral nutrition and/or parental nutrition support).”

The ESPGHAN also suggests that “a diagnosis of PIPO should be suspected in the following situations (2):

**FIGURE 1:**
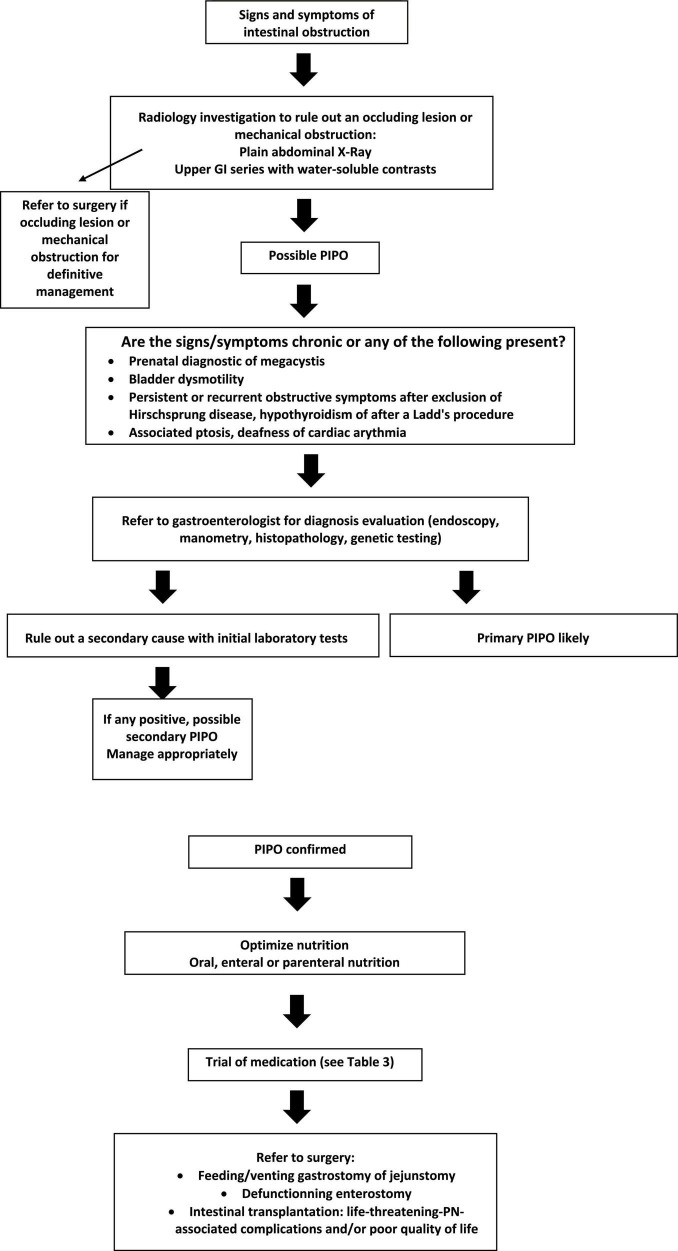
Algorithm for diagnosis and management of Pediatric Intestinal Pseudo-Obstruction.

• In all children presenting with symptoms of intestinal obstruction without an occluding lesion.
• In neonates with:
  ○ Prenatal diagnosis of megacystis/enlarged bladder.
  ○ Persistent or recurrent obstructive symptoms after exclusion of Hirschsprung disease and hypothyroidism.
  ○ Persistent vomiting after a Ladd procedure for malrotation.
  ○ Symptoms of intestinal obstruction associated with bladder dysmotility.

• In infants of children with:
  ○ Persistent or recurrent obstructive symptoms after exclusion of Hirschsprung disease.
  ○ Persistent vomiting/intestinal obstruction after correction of malrotation.
  ○ Symptoms of intestinal obstruction associated with:
    • Ptosis.
    • Deafness.
    • Abnormal cardiac rhythm/function.”

Initial work-up should be performed in a stepwise approach to rule out an occluding lesion, demonstrate GI neuromuscular involvement with a transit study, manometry and/or histopathology, find a genetic or metabolic abnormalities associated with PIPO and find a treatable systemic cause (24).

We suggest the following algorithm, adapted from the ESPGHAN algorithm, for the diagnosis and management of PIPO (2).

## Radiology

When PIPO is suspected, plain abdominal X-ray should be performed as a screening test to identify air-fluid levels and dilated bowel (2). However, contrast studies are required to eliminate an organic cause of obstruction since a plain radiograph cannot differentiate mechanical from functional obstruction. It can also rule out the presence of gut malrotation. Upper GI series with small-bowel follow-through studies should be done with water-soluble contrasts to avert possible formation of barium concretions in the colon. This is a universally available and non-expensive test but the large amount of contrast to ingest limits its use in PIPO. Contrast studies have been largely replaced in the recent years with enterography high-resolution CT or MRI which can more precisely expose intestinal adhesions and mechanical obstruction, and are now proposed as the first-line modalities (25).

Furthermore, cine-MRI is becoming known as a non-invasive, radiation-free diagnostic approach particularly in the pediatric population. It allows visual assessment of the entire small bowel, diameter measurements, displacement mapping, and GI tagging and shows decreased segmental contractility and small bowel motility in patients with CIPO (26, 27). Sato et al. evaluated recently specific CIPO detection parameters and concluded that when using these parameters, cine-MRI has the potential to help in the differential diagnosis of patients with intestinal dysmotility and could reflect the severity of the disease (28). Moreover, cine-MRI could be used to evaluate fasted and fed small bowel motility patterns as demonstrated by van Rijn et al. This study was the first to show an impaired response to food with MRI with the potential to use it for diagnosis of PIPO instead of manometry (29). Unfortunately, the major drawback in younger children is the need for general anesthesia which would affect the intestinal motility and would make it impossible to analyze the feeding patterns.

## Endoscopy

The main role of upper endoscopy in PIPO is to eliminate a mechanical obstruction of the upper small intestine and to obtain duodenal biopsies to rule out eosinophilic gastroenteropathy or celiac disease. Colonoscopy is rarely performed, but it can also rule out mechanical obstruction and help for decompression. Finally, Valli et al. recently proposed endoscopic full-thickness biopsy as a minimally invasive procedure with a good quantity of tissue for histopathological study in adult patients (30). There is currently no study in the literature evaluating the use of endoscopy to perform full-thickness biopsy in the pediatric population.

## Histopathology

The role of histopathology in the confirmation of PIPO is still debatable. It may provide useful information regarding the prognosis and possible specific management. The 2009 Gastro International working group proposed guidelines for histopathological methods and describing GI neuromuscular pathology (11, 31). Nevertheless, there remains a need for homogenized practice in histopathologic techniques (2, 11, 31).

Collection of gut-full thickness biopsy may reveal abnormalities in the smooth muscle celles, extrinsic or intrinsic neurons controlling gut functions and the ICC networks (4, 32). Recent studies in pediatrics using laparoscopic techniques for full-thickness biopsy have proven to be safe and effective for the diagnosis of a variety of GI problems, including PIPO (33). Rajan et al. performed duodenal and rectal endoscopic muscle biopsy on five pigs and demonstrated the technical feasibility, safety, and reproducibility of these procedures and the presence of myenteric neurons in all tissue samples (34). Emerging studies in adults show promising results with endoscopic-full thickness wall biopsy as a minimally invasive procedure with specimens of excellent quality and size for histopathological analysis and no immediate or long-term complications (30). More studies are needed in PIPO to prove the safety and efficacy of endoscopic-full thickness wall resection.

After reviewing 91 publications in the pediatric population regarding histopathological evaluation, ESPGHAN concluded that histopathological analysis should be performed in centers with expertise to undertake a full panel of neuromuscular labeling techniques in accordance with international guidance (2). This is particularly important for patients with conditions that could benefit from treatment with immunomodulation. Moreover, when therapeutic surgery is performed in children with PIPO, full-thickness biopsy should be obtained.

## Manometry

Antroduodenal manometry (ADM) is helpful in children with intestinal failure to define the pathogenesis, to enhance medical treatment, to decide if intestinal transplantation is indicated, and if so, what organs should be transplanted (35). The indications, technique, methodology, analysis, and clinical utility have been described elsewhere (35). ADM is particularly helpful in evaluation of the small intestine because it is nearly always affected. The recognition of a normal fasting pattern, the presence of normal phase III of the migrating motor complex (MMC), and the replacement of the MMC cycle by a fed pattern after a meal indicate a normal neuromuscular function (2). In patients with presumed intestinal failure, one of the primary contributions of ADM is to demonstrate normal physiology because it unequivocally eliminate PIPO (36). Children with symptoms of PIPO and a normal ADM should be evaluated for fabricated illness (37).

Antroduodenal manometry can help classify the types of pseudo-obstruction as myopathic or neuropathic. A neuropathic pattern has normal amplitude contractions but with disorganized and abnormal propagation of MMC. Other features of neuropathic PIPO are continued intestinal bursts of phasic pressure activity over 30 min, uncoordinated intestinal pressure activity or failure of the meal to produce a fed pattern. A myopathic pattern has preserved MMC but very low amplitude contractions (<20 mmHg) (35). However, low-amplitude contractions might only be a consequence of dilated bowel and need to be interpreted with caution (24).

Unfortunately, ADM has several limitations. The inter-observer agreement for identifying phase III of the MMC seems excellent, but the inter-observer agreement can vary for vague motor abnormalities (38). It is also invasive, expensive and not widely available (39). Furthermore, ADM can’t analyze the distal parts of the intestine because of the length of the catheter (28). Moreover, recent studies have demonstrated no correlation between specific manometric patterns and the histopathology (40). A 10-year study of patients with clinical diagnosis of CIPO who underwent ADM and full-thickness biopsy suggested that around 75% of patients with an abnormal ADM will have an abnormal intestinal histopathology, but histopathology could not be predicted by ADM findings (40). Another recent study aimed to elaborate a protocol for enhanced analysis of ADM contractile patterns and to explore whether it provided better correlation with histopathology (41). Forty pediatric patients were included in their study and they demonstrated that their newly developed enhanced analysis and associated score could differentiate PIPO and non-PIPO patients, but also apparent histopathological findings (41). It highlighted that patients with neuropathic histopathology may also have abnormal findings of phase I, not only phase III. Moreover, they could predict the requirement of parental nutrition with a higher score which confirms what was found in a previous study that demonstrated that children without a phase III during a 4-h ADM study were more likely to need parental nutrition (42). More studies on a larger population are required to corroborate their findings.

Finally, esophageal, colonic and anorectal manometry may be used to evaluate the magnitude of the disease in children with PIPO depending on the clinical presentation (2).

## Treatment and Outcome

The goal of treatment is to improve growth and nutrition, and optimize quality of life by limiting surgical interventions. A multidisciplinary team approach is recommended for the care of PIPO since it has a high number of comorbidities (2). Treatment includes nutrition, pharmacotherapy, and surgery.

Improving nutrition in children with GI dysmotility is difficult. Different approaches include oral feeding, enteral nutrition (EN) and parental nutrition (PN) depending on the patients’ tolerability. Children with PIPO often have malnutrition from malabsorption and food avoidance because of GI symptoms. Small, frequent meals low in fat and fiber are recommended as well as avoiding lactose-containing beverages and carbonated drinks (43). Over time, one third of patients will require total or partial PN, one third EN and the remaining will tolerate oral feeding (2).

Enteral nutrition can be given in bolus or continuous feeds depending, but gastric feeding should be considered first as it is more physiologic and easier. However, post-pyloric continuous feeds may be better tolerated in patients with PIPO (44).

Parental nutrition should be contemplated in patients with severe PIPO and not tolerating jejunal feedings or those who have contraindications for enteral feedings. Long-term PN leads to an important risk of complications: central line infection, central venous thrombosis, and liver disease (2) and 90% of patients with PIPO who die have one of these complications (5). Survival probability for patients with PN is reduced if they are <2 years old, have a very short bowel, have a stoma, myopathic PIPO and failure to resume oral feeding (45).

The objectives of pharmacological therapy are prevention of SIBO, improvement of GI motor function and management of pain. Unfortunately, there is no recommended drug treatment to ameliorate GI motility in almost all patients with PIPO (2). Most of the data on different drugs come from adult studies. Prokinetics, antiemetics, and antibiotics are the main treatment options in PIPO (2, 14). Prokinetics include 5-HT3 and 5-HT4 agonists (cisapride and prucalopride), cholinergics (bethanecol, neostigmine, pyridostigmine), macrolides (erythromycin, azithromycin), clavulanate-amoxicillin, octreotide and dopamine-2 receptor antagonists (domperidone and metoclopramide) (2, 4). The indications, mechanisms of action, suggested doses and their use in PIPO patients are described in Table 3 (2). These medications could be tried in PIPO patients at the discretion of the specialist caring for the patient.

**TABLE 3:** Prokinetic drugs used in Pediatric Intestinal Pseudo-Obstruction.

Medication	Dosage	Mechanism	Side effect	Contraindications/Interactions
**Prokinetics**				
Cisapride	0.2–0.3 mg/kg/dose 3–4 times a day, 30 min prior to a meal	5HT4 agonist with acetylcholine release in the gut 5HT3 antagonist	Nausea Vomiting Diarrhea Constipation Abdominal cramps Headache Cardiac arythmia Qtc interval prolongation	Absolute contraindication with other prokinetics (domperidone and metoclopramide) Absolute contraindication with cytochrome P4503A4 inhibitors: grapefruit juice, fluconazole, voriconazole, itraconazole, posaconazole, ketoconazole, erythromycin, clarithromycin, antiretroviral drugs Relative contraindication with known medications to prolong Qtc interval
Prucalopride	0.02–0.04 mg/kg/day max 2 mg daily	5HT4 agonist with acetylcholine release in the gut	Nausea Vomiting Diarrhea Abdominal pain Headache Little increase in pulse rate	↑ concentration of erythromycin ↓ biodisponibility of digoxin ↑ concentration of Prucalopride by ketoconazole, verapamil, cyclosporine, quinidine
**Cholinergics**				
Bethanecol	0.1–0.2 mg/kg/dose 3–4 times a day	Cholinergic acting on muscarinic receptor	Nausea Vomiting Diarrhea Abdominal pain Bronchial constriction Urinary frequency Miosis Lacrimation Flushing	↑ effect of cholinergic agents
Neostigmine	0.01–0.05 mg/kg/dose up to five times a day	Acetylcholinesterase inhibitor	Nausea Vomiting Diarrhea Abdominal pain Increased salivation Bradycardia Wheezing	Hypersensitivity to neostigmine or any component of the formulation Peritonitis or mechanical obstruction of the intestinal or urinary tract
Pyridostigmine	Start with 0.1–0.3 mg/kg/dose 2–3 times daily and increase as tolerated to a max of 7 mg/kg/day	Acetylcholinesterase inhibitor	Nausea Vomiting Diarrhea Abdominal pain Increased salivation Bradycardia Wheezing	Hypersensitivity of pyridostigmine or any component of the formulation Peritonitis or mechanical obstruction of the intestinal or urinary tract
**Macrolides**				
Erythromycin	3–5 mg/kg/dose 3–4 times daily Max 10 mg/kg/dose or 250 mg/dose	Motilin receptor agonist	Nausea Vomiting Abdominal cramps Abdominal pain Diarrhea Pseudomembranous colitis Cardiac arythmia Qtc interval prolongation Cholestasis Allergic reactions Pruritic rash	Absolute contraindication with cytochrome P4503A4 inhibitors: grapefruit juice, fluconazole, voriconazole, itraconazole, posaconazole, ketoconazole, erythromycin, clarithromycin, antiretroviral drugs
Azithromycin	10 mg/kg once daily	Motilin receptor agonist	Anorexia Arthralgia Anxiety Dizziness Dyspepsia Headache Hypoesthaesia Hearing loss (reversible) Paresthaesia	History of cholestasis or hepatic dysfunction associated with azithromycin
**Dopamine-2 receptor antagonists**				
Domperidone	0.1–0.3 mg/kg/dose to 0.6 mg/kg/dose 2–4 times daily 30 min prior to a meal Max 10 mg/dose	Dopamine-2 receptor antagonist Peripheral action (gut)	Nausea Diarrhea Abdominal cramps Qtc interval prolongation Cardiac arythmia Headache Hyperprolactinemia	Absolute contraindication with other prokinetics (cisapride, metoclopramide, prucalopride) Absolute contraindication with cytochrome P4503A4 inhibitors: grapefruit juice, fluconazole, voriconazole, itraconazole, posaconazole, ketoconazole, erythromycin, clarithromycin, antiretroviral drugs Relative contraindication with known medications to prolong Qtc interval
Metoclopramide	0.1–0.2 mg/kg/dose 3–4 times a day 30 min prior to a meal Max 10 mg/dose	Dopamine-2 receptor antagonist 5HT4 antagonist in the gut	Extrapyramidal reactions Late-onset dyskinesia Agitation Depression Drowsiness Fatigue Agranulocytosis Leucopenia Hyperprolactinemia Galactorrhea Gynecomastia	↑ concentration of cyclosporine, sirolimus, tacrolimus Close monitoring with other drugs known to cause extrapyramidal reactions
**Others**				
Octreotide	0.5–1 mg/kg subcutaneous once daily	Somatostatin analog	Nausea Vomiting Diarrhea Constipation Abdominal cramps Cholelithiasis Hepatitis Hyperglycemia Hypoglycemia Hypokalemia Bradycardia Cardiac arythmia Arthralgia Hypothyroidism Pancreatitis Muscular spasms	↓ concentration of cyclosporine Relative contraindication with known medications to prolong Qtc interval
Amoxicillin/clavulanate	20 mg/kg/day divided in two doses, up to antibiotic dose		Nausea Vomiting Diarrhea Dizziness Urticaria Hypersensitivity reaction Pseudomembranous colitis Hepatitis Headache	↓ efficiency of oral contraceptives ↑ levels of methotrexate

Recent studies have demonstrated the efficacy and safety of certain drugs used in adult CIPO for pediatric patients. Prucalopride has been shown to be safe and effective in reversing some of the symptoms of PIPO in one pediatric case series (46). In different case series, pyridostigmine was reported to be a good therapeutic option in PIPO with an easier administration than neostigmine (orally vs. intravenous) (47–50). Interestingly, a recent case report showed beneficial effects of cannabinoids in CIPO and could be considered in chronic abdominal pain non-responding to medical treatment and affecting quality of life (51). Further studies are required to consolidate these findings and to evaluate the safety in children.

A recent nationwide survey in Japan revealed that prokinetics, probiotics, laxatives and Japanese herbal medicine could be effective in some patients with PIPO (52). Furthermore, a recent pilot study demonstrated the safety of using fecal microbiota transplantation in adults with CIPO with improvement of symptoms, EN tolerance and oral feeding with no severe adverse events (53). Further studies are needed to confirm their findings, but it is promising.

A trial of immunotherapy like high doses of intravenous steroids, intravenous immunoglobulins, or apheresis, should be considered in patients with a suspected AGID, especially if antineuronal antibodies are detected (15, 17, 54). Improvement with immunotherapy could also aid in the diagnosis of AGID (15).

The main antibiotics that could be tried in SIBO are amoxicillin-clavulanate, metronidazole, trimethoprim-sulfamethoxazole, and aminoglycosides. Recent adult studies have shown clinical efficacy with rifaximin which has minimal systemic absorption and lower bacterial resistance (10). Repeated and prolonged use of antibiotics is not recommended, especially in children.

The role of surgery in PIPO is still controversial. Only necessary interventions should be performed given the potential risk of adhesions, prolonged ileus and deterioration of bowel function (2). The main procedures are palliative and include feeding/venting gastrostomies and jejunostomies and defunctionning enterostomy (ileostomy and colostomy) (2, 4). The aim is to decompress the bowel to make it work at its full potential. Recent studies evaluated the implementation of devices to provide electrical pacing to the GI musculature, but more studies are needed to confirm its safety and efficacy in PIPO patients (55, 56).

Intestinal transplantation (isolated or multi-visceral) is the only available life-saving therapy and should be considered in patients with life-threatening PN-associated complications [recurrent central line sepsis or loss of central line access, central line thrombosis or intestinal failure associated liver disease (IFALD)] and/or poor quality of life caused by repeated episodes of pseudo-obstruction with high risk morbidity and mortality (2, 10). With the simultaneous advances in post-operative management and surgical techniques, the outcomes and survival rates are better (89% at 1 year and 69% at 5 years) (57). Moreover, in the rare cases of MMIHS, the survival of these patients have significantly increased in the modern era of intestinal recovery and transplantation (100, 100, and 86% at 5, 10, and 20 years) (58).

## Conclusion

Pediatric Intestinal Pseudo-Obstruction remains a clinical challenge and one of the most severe intractable disease in pediatric gastroenterology with a marked impairment of GI motility. With the advances in clinical diagnosis and surgical techniques, the future of PIPO may be more promising. Firstly, less-aggressive investigations, like cine-MRI and maybe even endoscopic approaches for full-thickness biopsy for histological analysis, should facilitate the investigation of PIPO patients. Secondly, with more histopathological analysis performed in PIPO patients, we may be able to better understand the aetiopathogenesis of PIPO, and therefore identify novel targeted therapeutic options. Thirdly, in the current era of genetic sequencing, genetic studies are more informative and can help in therapeutic management. The coming years will definitely lead the way to more accurate diagnosis of PIPO, a better understanding of the etiologies and hopefully targeted therapeutic options.

## Data Availability Statement

The original contributions presented in the study are included in the article/supplementary material, further inquiries can be directed to the corresponding author.

## Author Contributions

M-CT reviewed the literature and wrote the first version of the manuscript. Both authors reviewed the final version of the manuscript, contributed to the article, and approved the submitted version.

## Conflict of Interest

The authors declare that the research was conducted in the absence of any commercial or financial relationships that could be construed as a potential conflict of interest. The handing editor TO declared a past co-authorship with CF.

## Publisher’s Note

All claims expressed in this article are solely those of the authors and do not necessarily represent those of their affiliated organizations, or those of the publisher, the editors and the reviewers. Any product that may be evaluated in this article, or claim that may be made by its manufacturer, is not guaranteed or endorsed by the publisher.
